# Directional interlayer spin-valley transfer in two-dimensional heterostructures

**DOI:** 10.1038/ncomms13747

**Published:** 2016-12-14

**Authors:** John R. Schaibley, Pasqual Rivera, Hongyi Yu, Kyle L. Seyler, Jiaqiang Yan, David G. Mandrus, Takashi Taniguchi, Kenji Watanabe, Wang Yao, Xiaodong Xu

**Affiliations:** 1 Department of Physics, University of Washington, Seattle, Washington 98195, USA; 2 Department of Physics and Center of Theoretical and Computational Physics, University of Hong Kong, Hong Kong, China; 3 Materials Science and Technology Division, Oak Ridge National Laboratory, Oak Ridge, Tennessee 37831, USA; 4 Department of Materials Science and Engineering, University of Tennessee, Knoxville, Tennessee 37996, USA; 5 Department of Physics and Astronomy, University of Tennessee, Knoxville, Tennessee 37996, USA; 6 Advanced Materials Laboratory, National Institute for Materials Science, Tsukuba, Ibaraki 305- 0044, Japan; 7 Department of Materials Science and Engineering, University of Washington, Seattle, Washington 98195, USA

## Abstract

Van der Waals heterostructures formed by two different monolayer semiconductors have emerged as a promising platform for new optoelectronic and spin/valleytronic applications. In addition to its atomically thin nature, a two-dimensional semiconductor heterostructure is distinct from its three-dimensional counterparts due to the unique coupled spin-valley physics of its constituent monolayers. Here, we report the direct observation that an optically generated spin-valley polarization in one monolayer can be transferred between layers of a two-dimensional MoSe_2_–WSe_2_ heterostructure. Using non-degenerate optical circular dichroism spectroscopy, we show that charge transfer between two monolayers conserves spin-valley polarization and is only weakly dependent on the twist angle between layers. Our work points to a new spin-valley pumping scheme in nanoscale devices, provides a fundamental understanding of spin-valley transfer across the two-dimensional interface, and shows the potential use of two-dimensional semiconductors as a spin-valley generator in two-dimensional spin/valleytronic devices for storing and processing information.

## Abstract (web summary)

Van der Waals heterostructures offer a platform for harnessing the spin-valley degree of freedom for information processing. Here, the authors transfer optically generated spin-valley polarization from one layer to another in a two-dimensional molybdenum diselenide–tungsten diselenide heterostructure.

Spin initialization is a crucial operation for spintronic devices which require a net spin polarization for reading, writing and transferring information^1^. Two-dimensional semiconductors, such as monolayer MoSe_2_ and WSe_2_, have recently emerged as a new spin/valleytronic platform^2^,^3^,^4^,^5^. Their inversion-asymmetric honeycomb lattice structures give rise to two energy degenerate but inequivalent (+K and −K) momentum-space valleys, forming a pseudospin system analogous to real electron spin^6^. Due to strong spin-orbit coupling, the valley pseudospin is locked to the real spin orientation^6^. Since flipping an electron spin requires a simultaneous flip of a valley pseudospin, free carrier spin-valley polarization at the band edge is expected to be robust and long lived, which has recently been measured to be on order of 1–100 ns (refs 7, 8). Large spin-valley polarizations associated with excitons have been generated by circularly polarized light excitation through a valley dependent optical selection rule^3^,^6^,^9^,^10^,^11^. However, excitonic spin-valley polarization in a monolayer does not last long compared with free carriers due to the picosecond timescale of the valley exciton depolarization time, which arises from the electron–hole exchange interaction^3^,^12^,^13^,^14^ and the ultrafast decay time of the exciton itself^15^,^16^. In addition, it is not clear how to exploit a monolayer system as a spin generator to supply optically generated spin-valley polarization to a different physical system.

2D semiconductor heterostructures formed by stacking two monolayers on top of each other can be designed to realize new spin-valley systems with important advantages over individual monolayers. It has been established that WX_2_–MoX_2_ (where X=S, Se) heterostructures have a type-II band alignment^17^,^18^, which leads to ultrafast charge transfer between layers and tunable photodetectors^19^,^20^. Such spatial separation of electrons and holes suppresses ultrafast electron–hole recombination^21^,^22^,^23^,^24^ and their exchange interaction^21^,^22^,^23^,^24^,^25^,^26^, both of which limit the practical application of optical spin-valley orientation in monolayers^3^,^27^,^28^. Very recently, helicity-dependent photoluminescence (PL) measurements of interlayer excitons revealed spin-valley polarization lifetimes exceeding tens of nanoseconds^26^, showing that the spatial separation of electrons and holes indeed provides a powerful approach towards practical spin-valleytronics. However, interlayer exciton effects were accompanied by complicated electron–hole relaxation pathways and the effect of the twist angle between the two layers^25^, which complicate the quantitative analysis of spin-valley polarization from the polarization resolved interlayer exciton PL. In addition, interlayer exciton PL studies were limited to small twist angle samples only, because the electron–hole momentum mismatch in large twist angle heterostructures strongly suppresses interlayer exciton light emission. All of these limitations obscured a clear understanding of the unique spin-valley properties of 2D semiconductor heterostructures, especially the transport of spin-valley polarized free carriers across the 2D layer interface.

In this work, by applying polarization resolved non-degenerate nonlinear optical spectroscopy, we provide a direct probe of interlayer spin-valley polarization transfer in a model 2D heterostructure with varying twist angles formed by monolayer MoSe_2_ and WSe_2_. By optically exciting an intralayer exciton spin-valley polarization in one layer and probing the intralayer neutral and charge excitons in different layers, we demonstrate that the subsequent interlayer charge transfer is directional and conserves spin, that is, spin polarization transfer leads to polarized hole spins in WSe_2_ and electron spins in MoSe_2_ (Fig. 1a). We find that the spin-valley polarization transfer has only a weak dependence on twist angles in the heterobilayer. Our results realize directional pumping of spin-valley polarized carrier spins into individual layers of a 2D heterostructure by harnessing the coupled spin-valley physics of the constituent monolayers^6^.

## Results

### Sample fabrication and electronic structure

**Figure 1:**
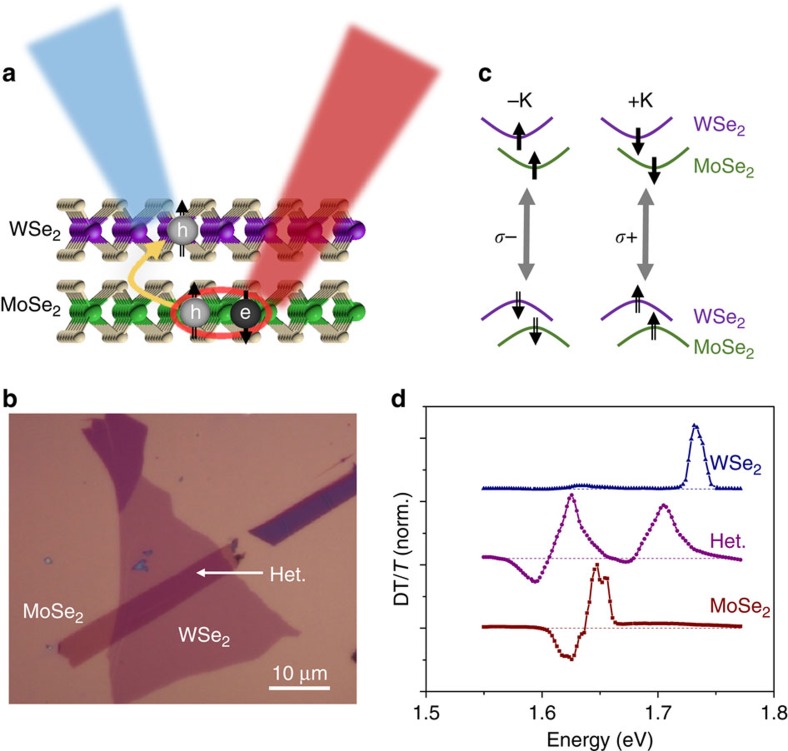
Interlayer spin-valley physics. (**a**) Depiction of the experiment. Spin-valley polarized excitons are resonantly injected in the MoSe_2_ layer with a polarized laser (red). The hole transfers to the WSe_2_ layer where its spin-valley polarization is measured with another polarized laser (blue), resonant with the WSe_2_ excitons. The black arrows depict the real spin of the electrons and holes. (**b**) Optical microscope image of a MoSe_2_–WSe_2_ heterostructure (Het.) on SiO_2_, showing the different sample regions. (**c**) The 8-band model of the +K and –K valleys for a nearly aligned MoSe_2_–WSe_2_ heterostructure, showing the valley dependent optical selection rules (*σ*± for ±K valley) and real spins (black arrows) for electrons and holes. (**d**) Degenerate DT spectra from different sample regions for a MoSe_2_–WSe_2_ heterostructure on sapphire, which are normalized and stacked for comparison. The dashed lines correspond to DT/*T*=0 for each spectrum. Due to the small isolated WSe_2_ area used in the DT study, the laser beam could not completely avoid the heterobilayer region, which results in the artifact of small positive signal at MoSe_2_ exciton energy on the WSe_2_ sample region.

The MoSe_2_–WSe_2_ heterostructures were fabricated from independently isolated, exfoliated monolayers (see Fig. 1b). To investigate the effect of heterostructure twist angle, we first measured the crystal axes of individual monolayers by polarization resolved and phase-sensitive second-harmonic generation spectroscopy^29^,^30^,^31^,^32^ (see Supplementary Fig. 1 and Supplementary Note 1). The monolayers were then assembled into heterostructures using a dry transfer stamping technique^33^ with known twist angle. Results from heterostructures with non-zero twist angels are presented in Supplementary Note 2 and Supplementary Figs 2 and 3. The sample in the main text has a twist angle near 0°, where the valleys from the different layers are nearly aligned in momentum space (Fig. 1c). The lowest conduction band is located in the MoSe_2_ and the highest valence band in WSe_2_. Within each monolayer, *σ*± circularly polarized light couples to transitions in the ±K valley only. The high quality of our heterostructure was confirmed by observing a strong PL quenching of the intralayer excitons, and the observation of interlayer excitons (see Supplementary Fig. 4), where Coulomb-bound electrons and holes are localized in opposite layers^24^.

### Nonlinear excitonic response of the heterostructure

We first determined the energy position of intralayer excitons by performing energy resolved continuous-wave differential transmission (DT) or differential reflection (DR) spectroscopy^34^. This is a two beam pump-probe technique which measures the difference of the probe transmission or reflection when the pump is on and off. The experiments in the main text were all performed on the same heterostructure mounted on sapphire. Additional measurements were performed on different heterostructures on SiO_2_ substrates and are in qualitative agreement with the data presented in the main text (see Supplementary Note 2). The experiments were performed at 30 K, unless otherwise specified.

The degenerate DT spectrum of a heterostructure is shown in Fig. 1d with cross-circularly polarized pump and probe. Compared with the DT spectrum from individual monolayers, we see the intralayer exciton resonances in the heterostructure are consistent with the spectral positions of isolated monolayers with a ∼20 meV redshift and broader linewidth. We attribute the ∼20 meV redshift to a reduction in the intralayer exciton bandgaps due to the coupling between layers. The linewidth broadening is attributed to the charge transfer between the layers, which leads to an extra relaxation channel for the intralayer excitons^35^. The resonance line shapes of the degenerate DT spectrum consist of a pump-induced increase to the probe transmission at high energy and a pump-induced absorption at low energy. Note that the low-energy pump-induced absorption feature is stronger for the MoSe_2_ layer compared with WSe_2_. We attribute this difference to the different oscillator strengths of different charged exciton species in each layer (see Supplementary Note 3).

### Demonstration of interlayer charge transfer

**Figure 2:**
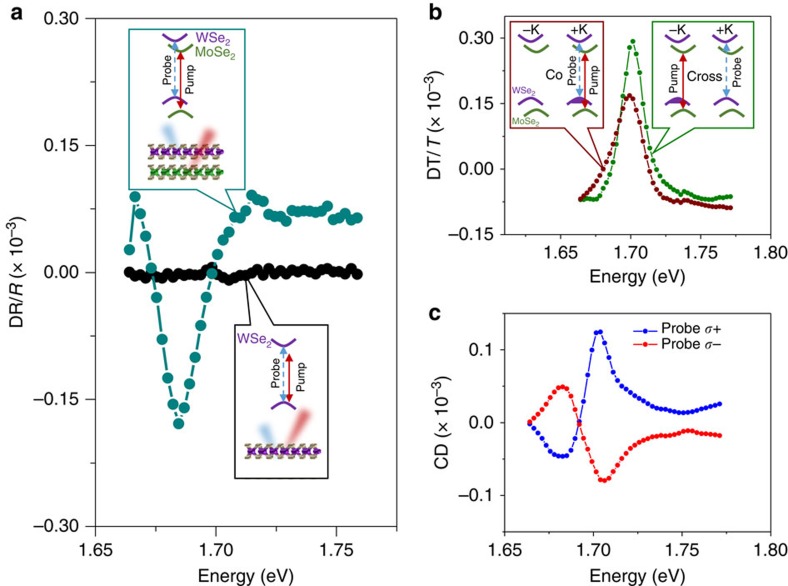
Interlayer hole spin-valley polarization transfer. (**a**) Non-degenerate DR of a MoSe_2_–WSe_2_ heterostructure, and an isolated WSe_2_ region on the sample. When pumping on the lower energy MoSe_2_ exciton resonance (1.621 eV), there is a strong DR response corresponding to the WSe_2_ exciton (dark cyan), whereas the isolated WSe_2_ monolayer shows negligible DR response (black). Co-circularly polarized pump and probe is shown. The insets depict the pump-probe scheme. The pump is shown as a solid red line, and the probe is the dashed blue line. DR data were measured at 50 K. (**b**) Co- (burgundy) and cross- (green) circularly polarized DT spectra of the WSe_2_ exciton resonances, when pumping the low-energy MoSe_2_ exciton resonance at 1.621 eV. The insets show the pump and probe scheme, where the band filling of the WSe_2_ valence is shown. The line shapes are discussed in the text. (**c**) Pump-induced CD of the WSe_2_ exciton resonances when pumping MoSe_2_ at 1.621 eV. CD highlights the differences between co- and cross-polarized DT responses. As expected, the sign of the CD response flips with probe (or pump) helicity.

To establish interlayer carrier transfer, we performed two-colour non-degenerate DR and DT measurements. Both types of measurements were performed on the same sample and the data are qualitatively similar. We use the DT data exclusively in curve fitting to avoid the interference effects that arise from the substrate reflection in the DR measurements. Figure 2a shows the DR spectrum with co-circularly polarized pump and probe, where the pump is resonant with the lower energy MoSe_2_ exciton at 1.621 eV while the probe laser scans over the WSe_2_ exciton resonance near 1.68 eV. The green curve shows an enhanced DR response from the heterostructure region. In comparison, the black curve shows the DR response when both pump and probe are focused on an isolated monolayer WSe_2_ region which shows a negligible DR response when the pump energy is fixed at the MoSe_2_ exciton resonance. In the heterostructure, since the MoSe_2_ exciton has lower energy than WSe_2_, the observed DR response near the WSe_2_ exciton when pumping the MoSe_2_ exciton resonance is unlikely from the energy transfer from MoSe_2_ exciton. Rather, it is a result of charge transfer from MoSe_2_ to WSe_2_. Specifically, the hole is transferred from the MoSe_2_ valence band to the WSe_2_ valence band due to the type-II band alignment.

### Demonstration of interlayer spin-valley polarization transfer

Interlayer spin-valley transfer was then investigated by performing polarization resolved DT experiments which measure the pump-induced circular dichroism (CD). The pump laser polarization and energy were chosen to only excite valley polarized excitons in the MoSe_2_ layer. The DT spectrum was measured for both co- (burgundy curve) and cross- (green curve) circularly polarized configurations for the probe scanning through the WSe_2_ excitons (Fig. 2b). The CD can be defined as the difference between the cross- and co-polarized DT spectra for either fixed pump or fixed probe polarization. Both yield similar results. For the convenience of our experimental configuration, we choose to fix the probe helicity while switching the pump helicity, that is, 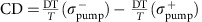, where the subscript denotes the pump beam, and *T* is the probe transmission. As shown in Fig. 2c, the sign of the pump-induced CD response reverses for opposite probe helicities. The observed CD demonstrates a valley population imbalance, that is, the creation of spin-valley polarization in WSe_2._ We attribute this population imbalance to the pumping of polarized hole spins as depicted in Fig. 1a. Circularly polarized excitation resonantly pumps spin-valley polarized excitons in the MoSe_2_ layer, about 60 meV below the WSe_2_ exciton energy. The spin polarized hole then transfers to the WSe_2_ +K valence band, which gives rise to hole spin-valley polarization in WSe_2_ and electron spin-valley polarization in MoSe_2_. The observation of the CD response supports this picture.

**Figure 3:**
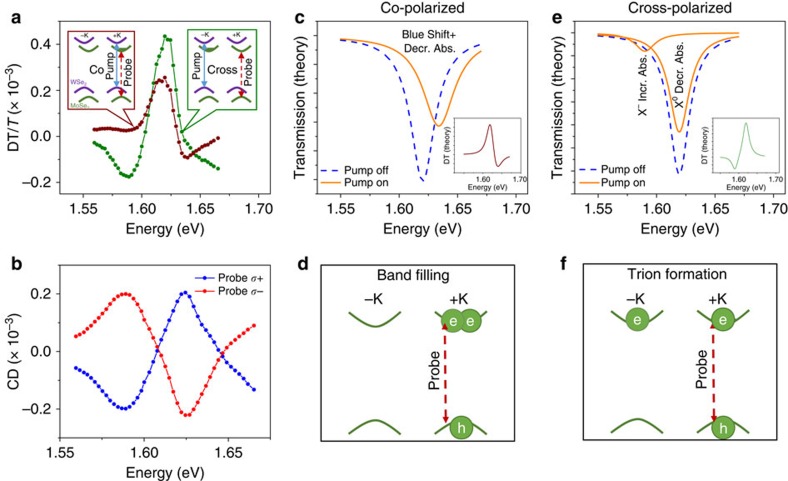
Interlayer electron spin-valley polarization transfer. (**a**) Co- (burgundy) and cross- (green) circularly polarized DT spectra of the MoSe_2_ exciton resonances, when pumping the higher energy WSe_2_ exciton resonance at 1.710 eV. The insets show the pump and probe scheme, where the pump is the solid blue line, and the probe is the dashed red line. Following the interlayer transfer of photo-excited electrons from WSe_2_ to MoSe_2_, spin-valley polarized electrons are pumped into the MoSe_2_ layer. The band filling of the MoSe_2_ conduction band is shown. (**b**) The pump-induced CD of the MoSe_2_ exciton resonances when pumping WSe_2_ at 1.710 eV flips sign with probe (or pump) helicity. (**c**–**f**) Theoretical explanations of the DT line shapes. (**c**,**d**) For co-polarized pump and probe, the polarized electrons populate the same valley that the probe measures. The dominant effect is a band filling effect, so that when the pump is on (orange curve in **c**), the resonance is blue shifted and the exciton absorption is partially saturated, relative to the pump off case (blue dashed curve in **c**). In this co-polarized configuration, a charged exciton cannot form due to Pauli blocking. (**e**,**f**) For cross-polarized pump and probe, the polarized electrons populate the opposite valley that the probe measures. The dominant effect is charged exciton (X^-^) formation, so that when the pump is on (orange curve in **e**), the transmission is decreased at the X^-^ resonance, and increased at the neutral exciton (X^o^) resonance, relative to the pump off case (blue dashed curve in **e**). The insets of **c**,**e** show the difference between the modelled pump on (orange) and pump off (blue dashed) curves, corresponding to the theoretical DT spectra.

We also demonstrate electron spin transfer from the WSe_2_ to the MoSe_2_ layer by resonantly pumping a WSe_2_ spin-valley polarization and probing the MoSe_2_ excitons (Fig. 3). Similar phenomena, including the pump-induced CD is observed, whose sign depends on probe helicity. Since the WSe_2_ exciton has higher energy than MoSe_2_, the observed CD will have two contributions. One is due to the electron spin transfer from the WSe_2_ to the MoSe_2_ conduction band. The other is from the above resonance optical excitation of valley-polarized excitons directly in the MoSe_2_. To distinguish these two effects, we measured the DR response on the heterostructure when pumping at the WSe_2_ resonance, and then repeated the measurement on the isolated monolayer MoSe_2_ region of the same sample (Supplementary Fig. 5). We observe a threefold enhancement of the DR response on the heterostructure region, which shows that electron transfer from the WSe_2_ to the MoSe_2_ dominates the DR response. We note that since the electron and hole spin are separated in opposite layers, the exchange interaction between the electron and hole spins is strongly suppressed. This will give rise to a long polarization lifetime^26^ and contributes to the enhanced DR response.

### Origin of the DT line shapes

We now turn to the discussion of the line shapes in the non-degenerate DT measurements (Figs 2b and 3a), which further support the picture of directional spin transfer. For simplicity, we focus on the explanation of data in Fig. 3a. Figure 3c–f illustrate the origins of the line shapes by pumping at the WSe_2_ exciton resonance while probing the MoSe_2_ excitons. The DT spectra can be understood by taking the difference between the probe transmission spectrum with the pump on and off (solid orange and dashed blue curve of Fig. 3c,e). The co-polarized pump and probe (burgundy data) laser configuration is shown in the left inset of Fig. 3a. The inset depicts the pump (solid blue line) injecting +K polarized carriers in the WSe_2_ layer and the consequent electron transfer to the +K conduction band valley in the MoSe_2_ monolayer. The probe (dashed red line) measures the changes in transmission spectrum of the +K MoSe_2_ excitons. Figure 3c,d depict the effects that dominate the co-polarized DT response. Because the conduction band is partially filled, phase-space filling leads to a blue shift of the transmission resonance, and the neutral exciton (X°) oscillator strength is reduced (Fig. 3c). The inset to Fig. 3c depicts the DT signal calculated by taking the difference between the orange and dashed blue curves.

The cross-polarized pump and probe configuration (green data) is depicted in the right inset of Fig. 3a. Here, the pump (solid blue line) injects carriers into the −K valley of the WSe_2_ layer, and the subsequent electron transfer to the –K valley of the MoSe_2_. With this –K valley electron population, when the probe beam (dashed red line) excites electron–hole pairs in +K valley, negatively charged excitons (X^−^) can form (Fig. 3e,f). The cross-polarized DT spectrum can be understood by examining Fig. 3e, which shows the pump-induced changes to the cross-polarized probe transmission spectrum. Relative to the pump off case, a population of electrons in the –K valley decreases the +K cross-polarized probe transmission at X^−^ resonance due to the increases of X^−^ oscillator strength, and increases the transmission at the X° resonance due to the decrease of X° oscillator strength. The inset to Fig. 3e shows the corresponding cross-polarized DT spectrum. We note that the 30 meV energy separation between the peak and dip in both the cross-polarized DT spectrum (green curve of Fig. 3a) and the CD spectra (Fig. 3b) is consistent with the binding energy of X^−^, and therefore further supports the picture of directional electron spin-valley transfer from WSe_2_ to MoSe_2_.

## Discussion

We estimate the resulting spin-valley polarization of electrons in the MoSe_2_ layer by pumping the WSe_2_ resonance and comparing the relative co- and cross-circularly polarized DT responses of the X^−^ in the MoSe_2_ layer. It has been demonstrated both experimentally^4^ and theoretically^36^ that the X^−^ in MoSe_2_ is dominantly an intervalley charged exciton with the extra electron located in the lower conduction band of the opposite valley (see Supplementary Note 4 and Supplementary Fig. 6). The X^−^ formation and the magnitude of its corresponding DT signal measures the population of polarized electrons in the valley opposite the one being probed. Therefore, spin-valley polarization (*ρ*) in the MoSe_2_ layer resulting from interlayer spin-valley transfer can be estimated by *ρ*=
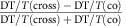
. Since the negative MoSe_2_ charged exciton resonance is spectrally isolated, it is not significantly influenced by signals arising from the other resonances (Fig. 3a). We fit single Lorentzians to the MoSe_2_ DT response near X^−^ (1.589 eV) for both co- and cross-circularly polarized pump and probe, and find that the ratio of the dip areas for the co-polarized response is 37% of the cross-polarized response (See Supplementary Fig. 7). This gives a 46% electron spin-valley polarization in the MoSe_2_ layer. This estimation is consistent with previous measurements of the interlayer exciton in helicity-dependent PL measurements, which was reported to be limited by depolarization of the monolayer exciton before interlayer transfer^26^.

When pumping the MoSe_2_ and probing the WSe_2_ excitons, the charged exciton feature is also clear in the CD response (Fig. 2c). Fitting the CD spectra with a difference of two Lorentzians, we find that the energy separation between the peak and dip is approximately 19 meV, consistent with the binding energy of positively charged excitons (X^+^) in WSe_2_ (refs 2, 3). This observation supports the picture of directional polarized hole spin transfer from MoSe_2_ to WSe_2_. However, due to the overlap of spectral features near the WSe_2_ positively charged exciton peak, we cannot accurately compare the co- and cross-circular DT responses of X^+^ to estimate a hole spin-valley polarization in the WSe_2_ layer.

We also performed measurements on additional samples with varying twist angles (Supplementary Fig. 2). There are fine spectral features distinct from near zero twist angle samples, which require a future systematic study. However, both the sign and signal amplitude of the CD spectra are consistent for all twist angles, which implies that spin-valley conserved interlayer charge transport is robust for different twist angles.

Our results demonstrate that spin-valley polarized carriers can be efficiently transferred between layers, providing a novel method for optically injecting long-lived and spin-valley polarized carriers in either layer of heterostructures with arbitrary twist angles. We expect this scheme could be especially useful in recent proposals that seek to use atomically thin-bilayer systems for spintronic or valleytronic applications^25^, or as a platform to investigate bosonic quasiparticle effects with spin structures^37^.

## Methods

### Sample fabrication

The heterostructures were assembled using a polycarbonate film dry transfer technique. Supplementary Note 1 contains the methods used to determine the crystal axes. The sample in the main text was encapsulated in 5–10 nm thick hexagonal boron nitride and mounted on a *c*-axis sapphire substrate to allow for optical transmission measurements.

### Nonlinear optical measurements

The data shown in the main text were measured in a cold-finger cryostat. Two continuous-wave tunable Ti:sapphire lasers (M^2^ SolsTiS) provided the pump and probe beams, which were each amplitude modulated with acousto-optic modulators at frequencies near 700 kHz. Both beams were actively intensity stabilized. A probe of 20 μW and a pump of 40 μW average power were used for all spectra in the main text. Polarizers and broadband waveplates were used to set the polarization of pump and probe, which were focused onto the sample with a microscope objective to a beam spot of ∼1 μm. The transmitted light was collected by a 15 mm spherical lens that was mounted in the cold finger of the cryostat. In the DR measurements, the reflected probe was collected with the objective. The pump beam was rejected with a cross-polarized set-up, or with a short or long pass filter. The probe was detected with an amplified silicon photodiode. The DT or DR signal was then measured with a phase-sensitive lock-in amplifier which was locked to the difference between the pump and probe modulation frequencies. The transmitted (*T*) or reflected (*R*) probe power was measured simultaneously with the DT or DR signal while the pump was modulated and used to normalize the DT/*T* or DR/*R* response.

### Data availability

The authors declare that all of the data supporting the findings of this study are available within the article and its Supplementary Information file.

## Additional information

**How to cite this article:** Schaibley, J. R. *et al*. Directional interlayer spin-valley transfer in two-dimensional heterostructures. *Nat. Commun.* **7,** 13747 doi: 10.1038/ncomms13747 (2016).

**Publisher's note**: Springer Nature remains neutral with regard to jurisdictional claims in published maps and institutional affiliations.

## Supplementary Material

Supplementary Information Supplementary Figures 1-7, Supplementary Notes 1-4 and Supplementary References.
