# Supplementary material for: Directional interlayer spin-valley transfer in two-dimensional heterostructures
Source: Nat Commun. 2016 Dec 14;7:13747. doi: 10.1038/ncomms13747 (PMC5171822; doi:10.1038/ncomms13747)
Supplement: Supplementary Information — Supplementary Figures 1-7, Supplementary Notes 1-4 and Supplementary References. [file ncomms13747-s1.pdf]

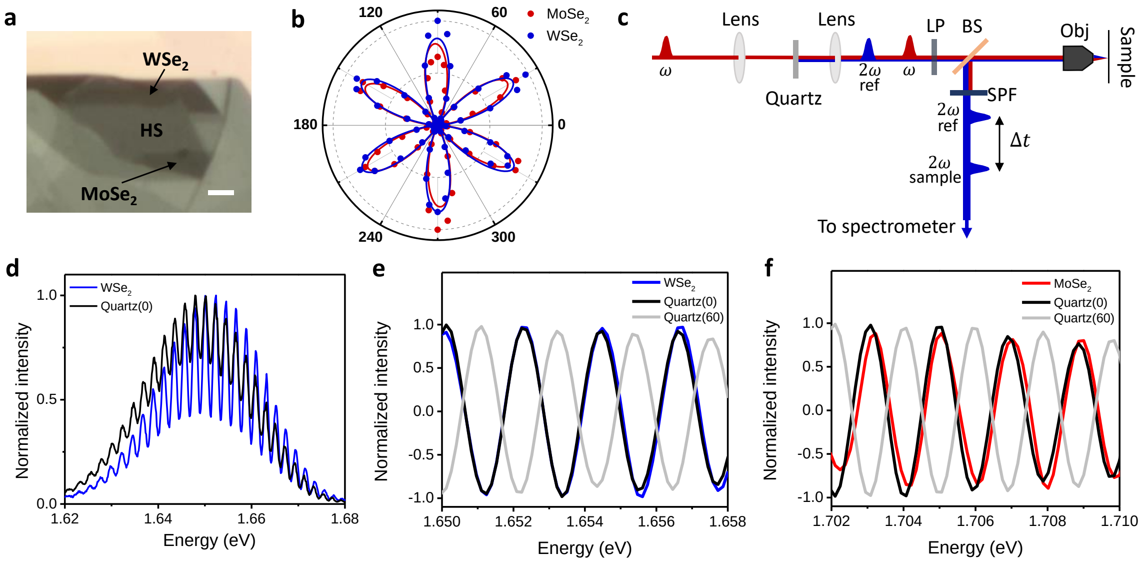

Supplementary Figure 1| Second-harmonic generation measurements. a, $MoSe_2$-WSe_2 heterostructure on sapphire from main text. The scale bar is 2 μm. **b,** SHG intensity parallel to the excitation polarization as a function of crystal angle for the monolayer $MoSe_2$ (red) and WSe_2 (blue) regions shown in **a**. The peaks of the lobes correspond to the armchair axes of the crystal. The relative angle between the lobe maxima is 1 ± 1°, meaning that the twist angle is close to 0° or 60°. **c,** Schematic of the phase-resolved SHG setup. LP, linear polarizer; BS, 50/50 beam splitter; SPF, short-pass filter; Obj, 50X objective. **d,** Second-harmonic interference spectrum from monolayer WSe_2 (blue) and from the front surface of z-cut quartz at 0° orientation (black). **e,** Close-up of the extracted SHG interference fringes for WSe_2 (blue) and two orientations of the quartz reference (black and gray), showing the SHG phase matches well with quartz(0) orientation compared to quartz(60). **f,** Same as in **e** but with $MoSe_2$ (red). This comparison shows the $MoSe_2$ phase agrees well with quartz(0) phase. Thus, the twist angle is near 0°.

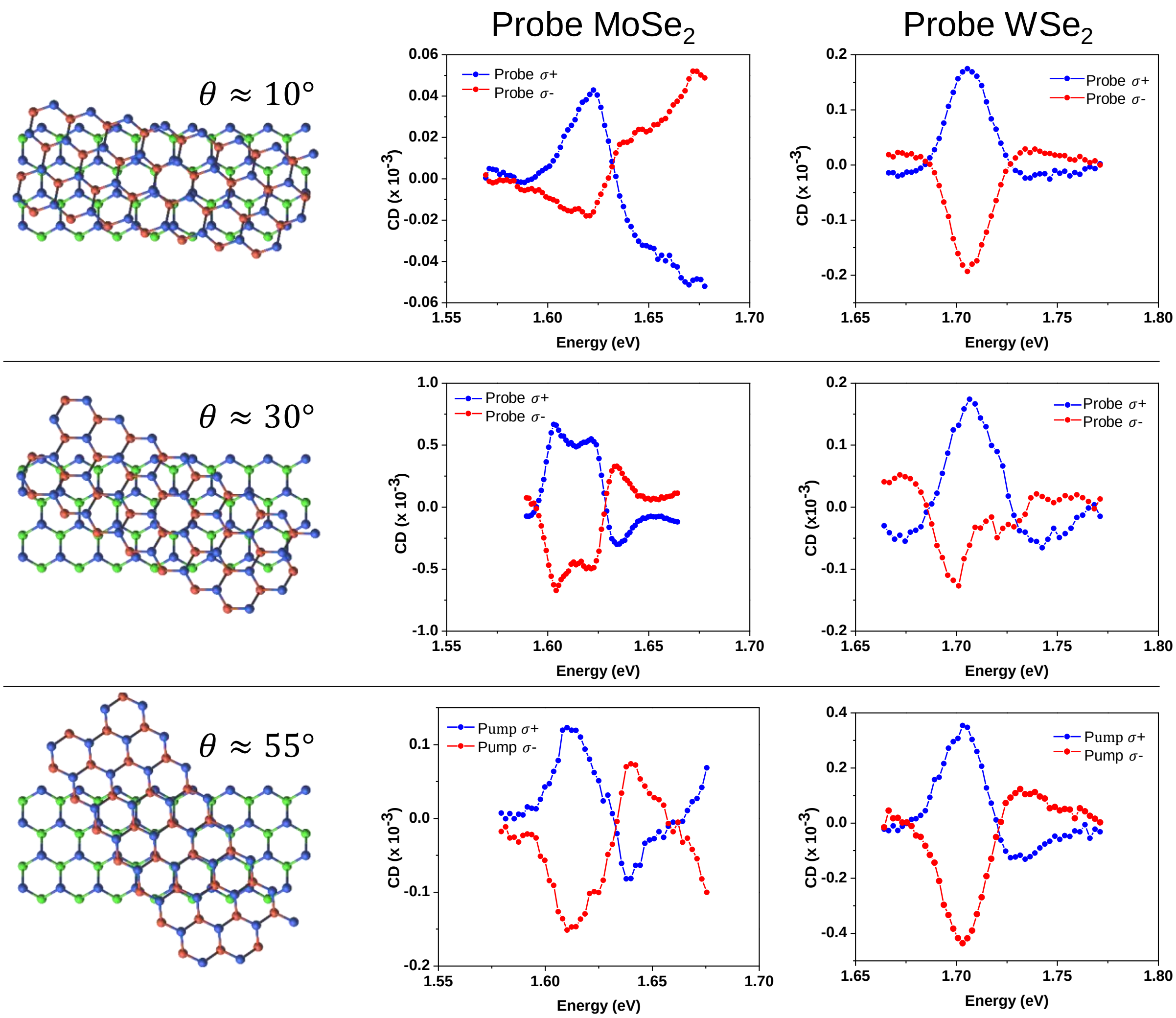

Supplementary Figure 2| Additional CD measurements on SiO_2 substrate samples. Each row corresponds to a different heterostructure sample with a different twist angle. The first column is a cartoon depicting the twist angle for each heterostructure. The blue atoms represent the selenium atoms and the red and green atoms represent the tungsten and molybdenum atoms, respectively. The second column shows the CD response from the $MoSe_2$ when pumping the WSe_2 near 1.71 eV. The third column shows the CD response from the WSe_2 when pumping the $MoSe_2$ near 1.63 eV. The pump and probe powers were in the 20-60 μW range. The labels for the data correspond to the pump and probe helicities respectively. For $\theta = 55°$, CD is shown varying the pump helicity instead of the probe helicity.

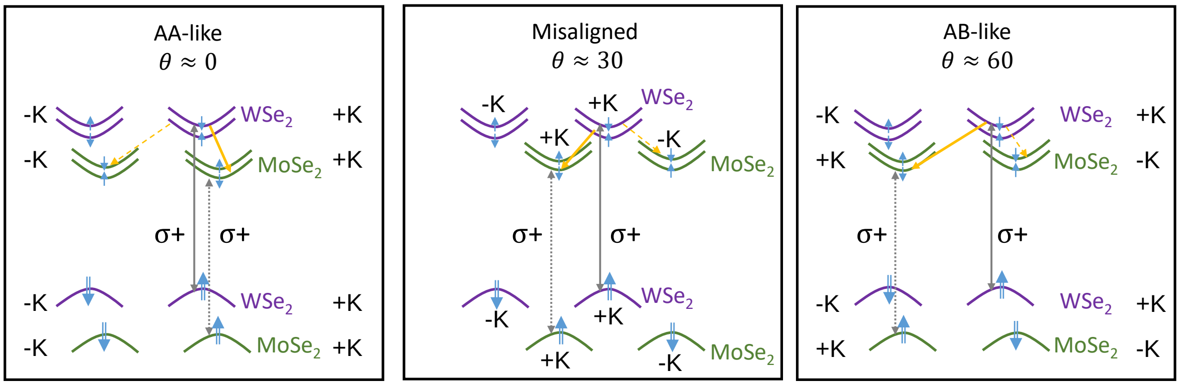

Supplementary Figure 3| Spin-valley transfer in twisted heterostructures. Electronic band structure for $MoSe_2$-WSe_2 heterostructures with different twist angles (θ) showing the spin split conduction bands. The WSe_2 bands are shown in purple and the $MoSe_2$ bands are green. The blue arrows depict the real spin for electrons in the conduction bands and holes in the valence band. The solid gray line depicts a pump laser exciting the +K valley of WSe_2. The dotted gray line depicts a probe laser probing the +K valley of $MoSe_2$. The yellow arrows show the interlayer transfer processes that conserve real electron spin. The solid yellow arrow shows transfer to the lowest energy $MoSe_2$ conduction band, whereas the dashed yellow arrow shows the transfer to the higher energy $MoSe_2$ conduction band. The CD data for different twist angles (Supplementary Figure 5) indicates that the spin transfer process is dominated by transfer to the lowest energy band for all twist angles (the transfer process shown by the solid yellow arrow). The effects arising from the upper conduction band are discussed below.

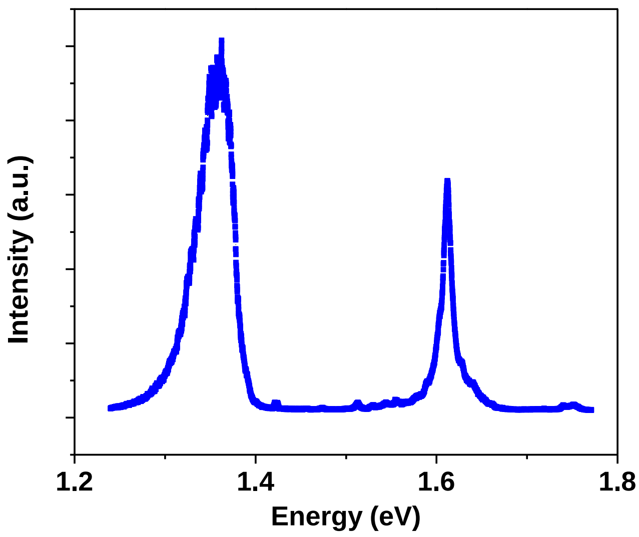

Supplementary Figure 4| Heterostructure photoluminescence. Photoluminescence spectrum of the heterostructure from the main text, recorded at 30 K with 30 µW excitation at 660 nm. Interlayer and $MoSe_2$ exciton peaks are observed centered at 1.355 eV and 1.612 eV respectively. The intralayer WSe_2 PL is negligible due to quenching.

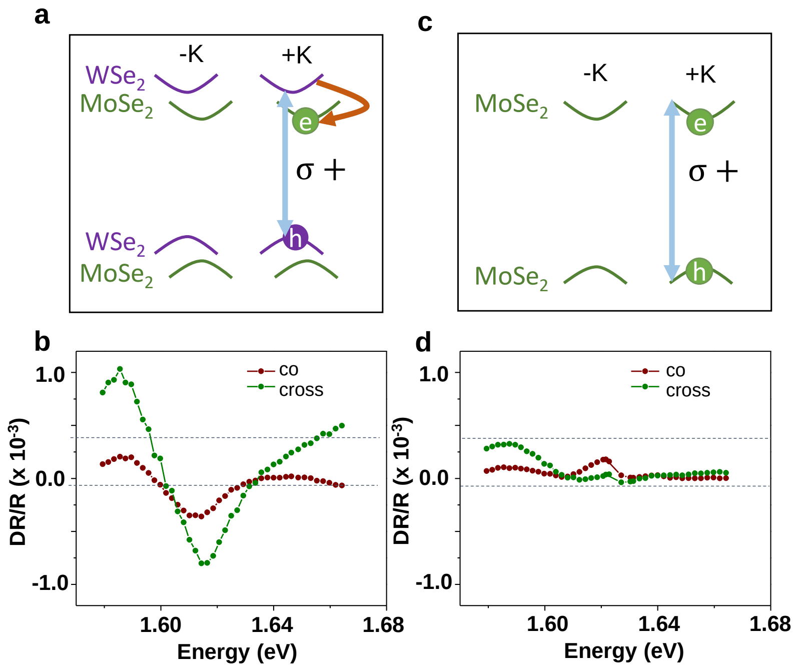

Supplementary Figure 5| Heterostructure enhanced spin-valley polarization in $MoSe_2$. a, In a heterostructure, +K spin-valley polarized electrons and hole are optically injected in the WSe_2 layer with circularly polarized light. The electron transfers to the +K conduction valley of the $MoSe_2$. **b,** DR spectra of the $MoSe_2$ exciton resonances when pumping WSe_2 at 1.687 eV, for co- (burgundy) and cross- (green) polarized pump and probe. **c,** In a monolayer $MoSe_2$ region, +K spin-valley polarized electrons and holes are directly optically injected in the $MoSe_2$ layer with circularly polarized light. **d,** DR spectra of the $MoSe_2$ exciton resonances when pumping at the WSe_2 resonance (1.687 eV) on an isolated $MoSe_2$ monolayer region for co-(burgundy) and cross-(green) polarized pump and probe. A comparison of **b** and **d** shows that the DR signal and the pumped-induced CD in **b** are primarily due to the interlayer electron transfer from the resonantly excited WSe_2 layer. Dashed lines are guides to the eye. DR measurements were performed at 50 K.

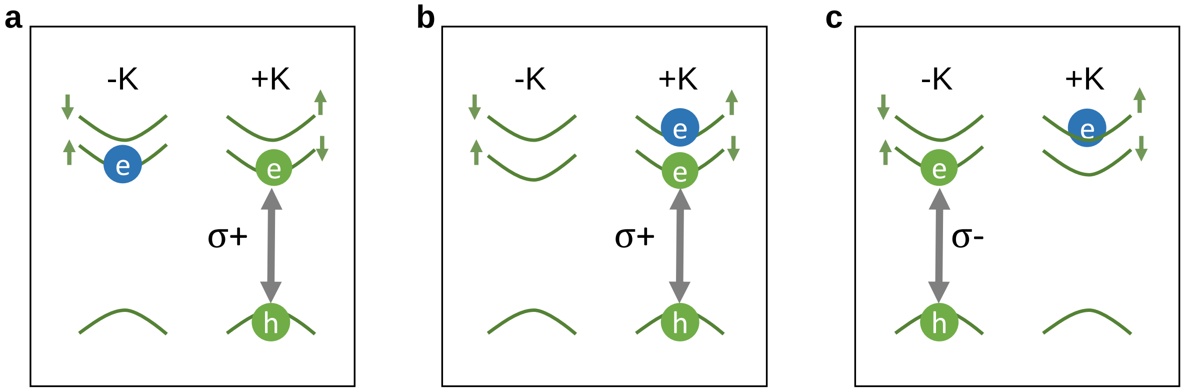

Supplementary Figure 6| $MoSe_2$ charged exciton configurations. a, The lowest energy negatively charged exciton has an intervalley configuration and consists of one electron in the lowest conduction band of one valley (-K shown) and the exciton in the opposite valley (+K shown). **b-c,** The higher energy charged exciton has the extra electron in the upper conduction band, which can have either an intravalley configuration (**b**) or an intervalley configuration (**c**). Note that the resonance energies for absorption or emission for all configurations are approximately equal, as discussed in S3. The arrow depicts the real spin of the electrons in each band.

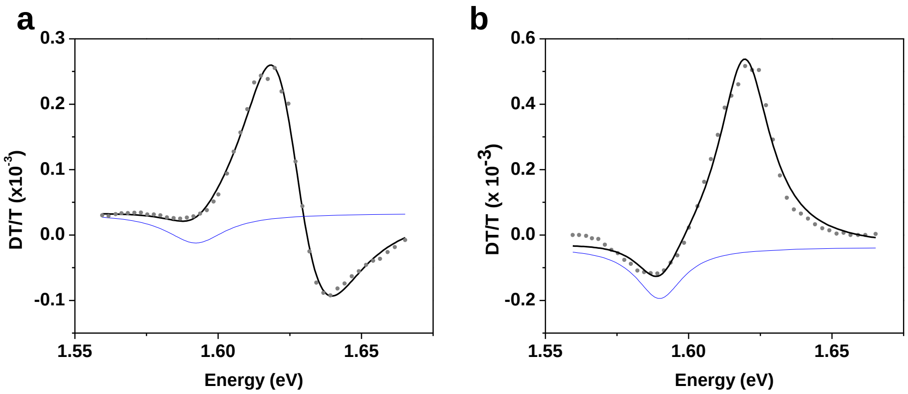

Supplementary Figure 7| Estimating the spin-valley polarization in $MoSe_2$. Detailed fitting to the data shown in Fig. 3a from the main text when pumping the WSe_2 heterostructure resonance near 1.710 eV, and probing the $MoSe_2$. We note that although we are plotting the same data as shown in Fig. 3a, a different aspect ratio is used to highlight the dip feature. By fitting the co- (**a**) and cross- (**b**) polarized DT spectra to a sum of three and two independent Lorentzians respectively, we compare the relative signal from the negative (dip) feature (shown in blue) centered near 1.59 eV, which corresponds to the energy of the negatively charged $MoSe_2$ exciton (X^-). Curve fitting reveals that the ratio of the areas for this dip feature is approximately 1: 0.37, yielding a spin-valley polarization of (1-.37)/(1+.37) = 46%.

Supplementary Note 1:

Measuring the Monolayer Crystal Axes by Polarization-Resolved and Phase-Sensitive Second-Harmonic Generation

To determine the relative crystal orientation between the monolayers in the heterostructure, we performed polarization-resolved and phase-sensitive second-harmonic generation (SHG) measurements. Here, we detail the measurement procedure for the heterostructure in the main text (Supplementary Figure 1a).

First, the orientation of the armchair axes of each monolayer was determined using co-linear polarization-resolved SHG as shown in Supplementary Figure 1b[1-4]. Since this technique is not sensitive to the phase of the SHG, it only determines the twist angle up to 60°. In this case, the relative angle between the lobe maxima is 1 ± 1°, which means the twist angle is close to 0° or 60°.

To resolve this ambiguity, we performed a SHG spectral interference experiment modelled after Ref 5-7. An optical parametric amplifier, pumped by an amplified mode-locked Ti:sapphire laser, launched tunable ~200 fs pulses at ω (typically ~0.83 eV) into the setup shown in Supplementary Figure 1c. The excitation was focused onto a z-cut quartz reference, which was oriented to generate horizontally polarized 2ω reference pulses (collinear to and co-polarized with the ω pulses). A time delay (Δt) was generated as the ω and 2ω pulses travelled through the dispersive optics in

the setup. A 50X objective focused the pulses onto the sample, generating an additional 2ω sample signal (time-delayed from the 2ω reference). The sample was oriented so an armchair axis, determined by the 6-fold pattern, was parallel to the 2ω reference and ω polarization. The back-reflected signals were diverted with a 50/50 beamsplitter through a 1 μm short-pass filter, into a spectrometer, and detected by a Si CCD. The time-delayed 2ω sample and reference signals produce spectral interference as shown in Supplementary Figure 1d. The period of the fringes is set by the time delay between the pulses (~2 ps) and the phase of the interference fringes is determined in part by the phase of SHG generated by the sample[5]. Therefore, this technique can distinguish between armchair axes 60° apart, which shows up as a π phase shift in the interference spectrum. The fringes can be separated from the broad background SHG through Fourier transforms, as shown in Supplementary Figure 1e-f[6,7].

Due the different resonances of $MoSe_2$ and WSe_2, it can be difficult to find a single excitation energy for direct comparison of the relative phase. To circumvent this issue, we also performed the interference experiment using a reference sample of z-cut quartz. When focusing on the front surface of the quartz, the allowed second-order susceptibility elements are the same as those of the monolayer MX_2. Thus, as before, we oriented the quartz with its "armchair" axis parallel to the excitation and reference pulses to get a similar interference spectrum (Supplementary Figure 1d, black). This was done for two orientations of the quartz, 60° apart. The WSe_2 and $MoSe_2$ interference experiments were then compared with the quartz experiment (Supplementary Figure 1e-f, respectively). In the case of the sample shown in Supplementary Figure 1, both the WSe_2 and $MoSe_2$ SHG fringes align well with the quartz(0) direction, which confirms the orientation is close to AA-like stacking.

Supplementary Note 2:

Effect of Twist Angle on the CD Response

Additional pump-induced circular dichroism (CD) measurements were repeated in the reflection geometry on three $MoSe_2$-WSe_2 heterostructures on SiO_2 substrates. The twist angles of these samples were not aligned ($\theta \neq 0$); however, we observed that both the sign and qualitative line shape (sign and amplitude) of the CD response were not significantly affected by the twist angle (Supplementary Figure 2).

It has been previously established that the heterostructure twist angle affects how the $MoSe_2$ and WSe_2 valleys line up in momentum space[4,8] (Supplementary Figure 3). Therefore, the independence of the CD response with respect to twist angle suggests that the interlayer spin transfer process does not require the valleys to line up in momentum space. Thus, we conclude that the interlayer CD response we measure arises from real spin conservation during the interlayer transfer process (as opposed to valley pseudospin conservation). In the simplest model, spin-polarized carriers transfer between layers and then relax into the lowest energy state in each layer while maintaining their spin polarization as depicted in Supplementary Figure 3. We note that this conservation of real spin polarization also leads to a valley polarization in each layer due to the spin-valley locking effect[9].

Supplementary Note 3:

Degenerate DT Line Shape and Doping Effects

In this note, we examine the degenerate DT spectrum (Fig. 1c) and discuss the effects of unintentional doping. In the heterostructure region, the $MoSe_2$ (WSe_2) resonance is fit by a difference of two Lorentzians which reveals a weaker dip feature centered at 1.594 eV (1.675 eV) and stronger peak feature centered at 1.625 eV (1.705 eV). For each resonance, the ~20-30 meV difference between the peak and dip is consistent the reported binding energies for charged excitons. We therefore attribute the positive DT signal peaks to reduced neutral exciton absorption and the negative DT signal dips to increased charged exciton absorption. Both effects can be explained by pump induced photo-doping which increases the oscillator strength for charged excitons and reduces the oscillator strength for neutral excitons.

We note that the low energy pump induced absorption feature is stronger for the $MoSe_2$ layer. We attribute this difference between the WSe_2 and $MoSe_2$ response to the different species of charged exciton for each layer. As discussed in the main text, the WSe_2 pump induced absorption arises from formation of positively charged excitons (X^+), whereas the $MoSe_2$ pump induced absorption arises from negatively charged excitons (X^-). One possibility is that the oscillator strength for the X^+ in WSe_2 is weaker than that of the X^- in $MoSe_2$, which is consistent with previous studies showing that the X^+ in WSe_2 has weaker PL[10]. We also note that PL measurements indicate that our $MoSe_2$ is weakly electron doped, whereas the WSe_2 is nearly intrinsic. However, since we are performing a differential measurement, we expect the effect of the background unpolarized doping to be small.

Supplementary Note 4:

Effect of the Upper Conduction Band in $MoSe_2$

In the main text, we use the relative amplitudes of the DT responses near the negatively charged exciton (X^-), to estimate the electron spin polarization in the $MoSe_2$ layer. The lowest energy X^- corresponds to an intervalley configuration where, for example, one electron is in the lowest conduction band of one valley (-K), and the exciton is in the other valley (+K), as depicted in Supplementary Figure 6a. An intervalley trion with two electron in the same valley is not allowed due to Pauli blocking. Therefore, a -K valley electron in the lower conduction band can only induce $\sigma+$ polarized optical absorption at the X^- resonance. The conduction band splitting has been calculated to be on order of 20 meV[11].

However, there is also the possibility of an X^- composed of an electron in the upper conduction band which can either be in an intravalley (Supplementary Figure 6b) or intervalley (Supplementary Figure 6c) configuration and couples to a $\sigma+$ or $\sigma-$ polarized photon. The photon energy is given by the X^- energy minus the upper conduction band electron energy, close to the lowest energy X^- optical absorption. We note that: (1) The upper conduction band electrons have higher energies, so they will relax to the lower conduction band with the same spin[12]. Thus, in the

steady state, the upper conduction band population is expected to be small. (2) A small electron population in the upper conduction band does not significantly affect our results. For example, if there is a small population of electrons in the upper conduction band, it increases the oscillator strength both for intravalley and intervalley charged excitons as shown in Supplementary Figure 6b-c. This population would lead to increased absorption for both co-polarized ($DT/T(Co)$) and cross-polarized ($DT/T(Cross)$) signals, which contributes to $DT/T(Cross) + DT/T(Co)$. But to lowest order, these two contributions are equal in magnitude, so they give rise to a negligible CD response which is proportional to the difference, $DT/T(Cross) - DT/T(Co)$. In this case, the value $\rho = \frac{DT/T(Cross) - DT/T(Co)}{DT/T(Cross) + DT/T(Co)}$ corresponds to a lower bound for the spin-valley polarization of the lower conduction band electron in the $MoSe_2$ layer.

Supplementary References:

1 Kumar, N. *et al.* Second harmonic microscopy of monolayer MoS_2. *Phys Rev B* **87**, 161403 (2013).

2 Malard, L. M., Alencar, T. V., Barboza, A. P. M., Mak, K. F. & de Paula, A. M. Observation of intense second harmonic generation from MoS_2 atomic crystals. *Phys Rev B* **87**, 201401 (2013).

3 Li, Y. *et al.* Probing symmetry properties of few-layer MoS_2 and h-BN by optical second-harmonic generation. *Nano Lett.*, 3329–3333 (2013).

4 Rivera, P. *et al.* Valley-polarized exciton dynamics in a 2D semiconductor heterostructure. *Science* **351**, 688-691 (2016).

5 Lepetit, L., Cheriaux, G. & Joffre, M. Linear techniques of phase measurement by femtosecond spectral interferometry for applications in spectroscopy. *JOSA B* **12**, 2467-2474 (1995).

6 Veenstra, K., Petukhov, A., De Boer, A. & Rasing, T. Phase-sensitive detection technique for surface nonlinear optics. *Phys Rev B* **58**, R16020-R16023 (1998).

7 Wilson, P., Jiang, Y., Aktsipetrov, O., Mishina, E. & Downer, M. Frequency-domain interferometric second-harmonic spectroscopy. *Opt. Lett.* **24**, 496-498 (1999).

8 Yu, H., Wang, Y., Tong, Q., Xu, X. & Yao, W. Anomalous light cones and valley optical selection rules of interlayer excitons in twisted heterobilayers. *Phys. Rev. Lett.* **115**, 187002 (2015).

9 Xiao, D., Liu, G.-B., Feng, W., Xu, X. & Yao, W. Coupled spin and valley physics in monolayers of MoS_2 and other group-VI dichalcogenides. *Phys. Rev. Lett.* **108**, 196802 (2012).

10 Jones, A. M. *et al.* Optical generation of excitonic valley coherence in monolayer WSe_2. *Nature Nanotech.* **8**, 634-638 (2013).

11 Liu, G.-B., Shan, W.-Y., Yao, Y., Yao, W. & Xiao, D. Three-band tight-binding model for monolayers of group-VIB transition metal dichalcogenides. *Phys. Rev. B* **88**, 085433 (2013).

12 Yang, L. *et al.* Long-lived nanosecond spin relaxation and spin coherence of electrons in monolayer MoS_2 and WS_2. *Nat Phys*, 830–834 (2015).
